# 616. Beyond the Burn: The Social Drivers and Cost of Extended Hospitalization in Burn Care

**DOI:** 10.1093/jbcr/irag033.430

**Published:** 2026-04-06

**Authors:** Adrienne E DeVault, Anjay Khandelwal, Ayla R Mapes

**Affiliations:** Akron Children's Hospital, Akron, OH; Akron Children's Hospital, Akron, OH; Akron Children's Hospital, Akron, OH

## Abstract

**Introduction:**

Burn patients often have a long length of stay that creates additional costs to both the patient and hospital system. While researchers have analyzed medical and surgical factors affecting LOS to improve prediction estimates, studies have not yet examined the specific social and economic complexities that interfere with discharge processes, especially after the patient has been medically cleared for discharge. We sought to explore factors impacting discharge among adult and pediatric patients medically ready for discharge, determine how many days patient stay was extended, and the determine impact on cost.

**Methods:**

We conducted a retrospective chart review for adult and pediatric patients admitted to a regional verified burn center over the past two years. From this group, we excluded patients who died in hospital or had a length of stay less than three days. The date that each that each patient was medically ready for discharge was determined by individual chart review, and patients discharged later than that date were identified, and descriptive statistics were examined. Actual hospital cost data including fixed and variable direct charges were determined.

**Results:**

Overall, 12.3% of all admitted patients were identified as having extended hospital stays due to social (non-medical) factors, of which 82.4% were adults. Overall, 38.2% of the sample were female, 73.5% were White, and 26.5% were Black.

Among pediatric patients, the mean age was 1.51 years (SD = 0.8), 39.3% female, and all cases involved scald burns. The average number of days the hospital admission was extended was 3.33 days (SD = 2.88). The most common etiology was the involvement of child protective services (CPS) (66.7%), primarily due to placement and custody issues.

Among the adult patients, the mean age was 59.93 years (SD = 16.82), 39.3% female, and 46.4% experienced flame burns. The average duration of extended hospitalization was 6.71 days (SD = 5.4). The top three contributing social factors along with number of days extended are presented in Table 1. Social support issues included challenges finding transportation or family needing time to prepare for care at home.

Actual hospital cost (not charges) for the additional length of stay in the hospital was estimated at $11 210 for each of the pediatric patients and $28 236 for each of the adult patients. A positive relationship between mental health disorders and extended length of stay was noted.

**Conclusions:**

Results highlight that CPS involvement and transferring care to specialized facilities often delays discharge despite patients being medically ready. This leads to increased waste and mismanagement of finite resources, which increases the cost burden for already vulnerable patient populations and the institution.

**Applicability of Research to Practice:**

Investigating the reasons behind non-medical extended lengths of stay for burn patients will pave the way for future research to explore potential solutions to this costly problem.

**Funding for the study:**

N/A.

